# Adolescent Girls’ Breast Self‐Examination Practices in Eastern Region of Ghana

**DOI:** 10.1155/tbj/5207043

**Published:** 2026-01-31

**Authors:** Stella Sagoe, Patricia Tsotsoo Clottey, Isaac Nyarko Kwakye, Emmanuel Lamptey, Sussana Sagoe, Thywill Amenuveve Degley, Ruth Nimota Nukpezah, Daniel Adom-Fynn

**Affiliations:** ^1^ Department of Nursing, Central University, Accra, Ghana, central.edu.gh; ^2^ Ear, Nose and Throat Department, University of Ghana Medical Centre, Accra, Ghana, ugmedicalcentre.org; ^3^ Department of Environment and Public Health, University of Environment and Sustainable Development, Somanya, Ghana, uesd.edu.gh; ^4^ School of Basic and Biomedical Sciences, University Health and Allied Sciences, Ho, Ghana; ^5^ Department of Public Health, Community Health Nursing Training School, Oda, Ghana; ^6^ School of Nursing, University of Development Studies, Temale, Ghana; ^7^ Department of General Studies, University of Environment and Sustainable Development, Somanya, Ghana, uesd.edu.gh

**Keywords:** adolescents, breast self-examination, knowledge, practices

## Abstract

**Background and Aims:**

Breast cancer incidence is rising globally, including in Ghana, making early detection vital. Breast self‐examination (BSE) is key to reducing morbidity and mortality. This study assessed BSE practices among adolescent girls in Ghana’s Eastern Region to inform prevention efforts.

**Methods:**

A cross‐sectional design was adopted, and the simple random sampling strategy was used to recruit 385 female students from Aburi Girls’ Senior High School. Data were collected using a structured questionnaire covering demographic information, BSE knowledge, and practices and were analyzed using SPSS Version 21. Descriptive statistics were used to summarize the data, while Pearson’s correlation assessed the association between knowledge and practice. Logistic regression was performed to determine the predictive effects of demographic variables on BSE practice.

**Results:**

Participants demonstrated moderate to high knowledge of BSE with mean scores ranging from 1.00 to 1.92, with strongest awareness of palpation techniques (mean = 1.92, SD = 0.272) but lower understanding of positional and visual inspection methods. Most respondents (67.3%) knew how to perform BSE, yet only 25.2% adhered to monthly timing guidelines. Hands (75%) and mirrors (25%) were commonly used, with 41.3% performing both palpation and observation. Knowledge and practice were positively correlated (*r* = 0.149, *p* = 0.003). Logistic regression identified age, educational level, knowing someone with breast cancer, and workshop attendance as significant predictors of BSE practice (*p* < 0.001).

**Conclusion:**

Educating adolescent girls on breast cancer and proper BSE practices is essential. Targeted interventions can strengthen knowledge and skills among young Ghanaian women, supporting early detection and broader prevention efforts.

## 1. Introduction

Breast cancer in women is a significant public health challenge and the leading cause of cancer‐related morbidity and mortality among women globally [1, 2]. This reflects disparities in early detection, treatment access, and health system capacity [3, 4]. Recent global cancer statistics indicate that breast cancer incidence is increasing more rapidly in developing regions [5]. In 2020, breast cancer accounted for 2.3 million new cases and 685,000 deaths worldwide, with 7.8 million women diagnosed within the previous five years [6]. That year, the disease constituted 29.5% of all cancer cases and 22.1% of cancer‐related deaths [7]. Projections suggest that cases and deaths will increase substantially by 2050 if current trends continue, particularly in low human development index (HDI) countries [3]. In Ghana, breast cancer accounts for 31.8% of all cancer cases, making it the most prevalent type of cancer in the country [7]. Alarmingly, the age of diagnosis can be as early as 14 years, with an increasing incidence observed among premenopausal women [8]. Reports indicate that the average age of breast cancer diagnosis in Ghana is between 40 and 50 years [9]. According to Osei‐Afriyie et al. [10], the aggressive nature of breast cancer in younger women, combined with limited access to early detection and treatment, exacerbates the disease’s impact. The lack of widespread screening programs and the presence of cultural and socioeconomic barriers further contribute to delayed diagnoses and poorer outcomes in Ghana.

Approaches to reduce cancer‐related suffering and mortality encompass the entire spectrum of cancer control, including prevention, early detection, treatment, and palliative care. Early detection of breast cancer is particularly critical, as it represents the most effective strategy for decreasing morbidity and mortality associated with the disease [11, 12]. The American Cancer Society recommends three primary screening methods: mammography, clinical breast examination, and BSE [9]. Although mammography is recognized as the most effective method for early detection, its high costs and limited accessibility in many developing countries render making BSE a more feasible and cost‐effective alternative [11, 12]. Approximately 70% of breast cancer‐related deaths are estimated to occur in low‐ and middle‐income countries (LMICs) [13]. This high mortality rate can be largely attributed to delays in diagnosis due to combination of myths, misconceptions, cultural beliefs, and inadequate diagnostic facilities [14].

BSE, a low‐cost method for women to inspect and palpate their breasts for changes, has been studied for earlier detection where formal screening (mammography) is limited. Systematic reviews and contemporary studies show that while awareness of BSE is high, regular monthly practice is low [15–17]. A pooled review by Seifu and Mekonen [17] found that many women have heard of BSE, but only one‐third or fewer perform it monthly in various settings. Student‐ and community‐based studies corroborate this trend, revealing that knowledge and exposure are common, but consistent practice and correct technique are less frequently reported [18, 19]. Among Jordanian students, medical workers, and women in health‐related fields, many are aware of BSE and breast cancer, yet they often lack a comprehensive understanding of screening methods and guidelines, leading to persistently low BSE practice [20, 21]. Clinical guidelines differ on the role of BSE; evidence suggests that routine instruction does not significantly reduce breast cancer mortality and may increase false‐positive results and unnecessary diagnostic procedures [22]. Consequently, many high‐income countries have excluded BSE from screening recommendations, focusing instead on breast awareness and timely clinical evaluation of symptoms [23]. Conversely, public health researchers argue that in low‐resource contexts, promoting breast awareness and teaching self‐examination techniques can improve early detection where mammography and clinical services are scarce [24]. In many sub‐Saharan African and resource‐limited settings, BSE is promoted as a practical early‐detection method due to limited or unaffordable access to mammography and clinical breast examinations [15, 17, 25]. A systematic review and meta‐analysis show that BSE is extensively studied across Africa, particularly where formal screening infrastructure is weak [17]. Community‐based findings from Ethiopia indicate that BSE is an inexpensive, simple technique that helps women familiarize themselves with their breast tissue and detect abnormalities without mammography services [26, 27]. A mixed‐methods study from Nigeria also highlights BSE as a viable early‐detection option in low‐resource environments where women face barriers to accessing mammography or routine clinical exams [28].

Studies indicate that breast cancer in adolescents and young women often presents at more advanced stages and with more aggressive characteristics compared to older women [29–32]. Data from Ghana show that women under 35 years frequently present with invasive carcinoma, indicating a trend of early‐onset disease with potentially worse prognosis [33–35]. Given that adolescence and young adulthood are formative periods when lifelong health habits and screening behaviors are established, targeted education and early detection practices, such as breast BSE, may be particularly important for this group. However, evidence regarding BSE knowledge and practice among adolescents and young women in Ghana’s Eastern Region remains limited. This study aims to explore the knowledge, attitudes, and practices (KAP) of BSE among adolescent girls, as well as examine the correlation between knowledge and practice of BSE, and the socio‐demographic predictors of BSE practice. By focusing on senior high school students in this region, this study seeks to address this gap and provide data on how awareness, attitudes, and screening behaviors intersect in the context of early‐onset breast cancer risk.

## 2. Materials and Methods

### 2.1. Research Design

The study used a cross‐sectional design to examine students’ knowledge and practices regarding BSE. This design allowed the researchers to gather data from the population at a specific moment in time [36] and is relevant for assessing the prevalence of disease, attitudes, and knowledge [37].

### 2.2. Research Setting

The study took place at Aburi Girls’ Senior High School in the Eastern Region of Ghana. This location was chosen to focus on female adolescents who will become mothers.

### 2.3. Population and Sample

The study population included all female students at Aburi Girls’ Senior High School, totaling 1800. The sample size was determined using Yamane’s [38] formula for known population size resulting in the selection of 385 students. Simple random sampling was employed, ensuring each member had an equal chance of selection [39]. Researchers used student lists from each class as the sampling frame, assigning each student an ID number on separate pieces of paper placed in a box. The papers were shuffled, and random numbers were drawn without replacement until the desired sample size of 385 participants was reached.

### 2.4. Inclusion Criteria

The study included all female students at Aburi Girls’ Senior High School, both boarding and day students from Forms 1, 2, and 3. Female students admitted to the hospital during the study were excluded.

### 2.5. Instrument

The study used a structured questionnaire divided into three sections. Section A includes demographic information such as age, gender, level of education, religion, history of breast cancer, knowing someone with breast cancer, and attendance at workshops, conferences, or seminars on breast cancer. Section B assesses the knowledge of the respondents on BSE, based on guidelines from Birhane et al. [12] and Mihret et al. [40]. Section C focuses on the practice of BSE among the students.

### 2.6. Pretesting

Pretesting was conducted among 30 students at Wesley Grammar Senior High School in Accra. Modifications were made to the questionnaires based on the responses to ensure accuracy and clarity. The Cronbach’s alpha reliability test was conducted in SPSS, resulting in an overall reliability coefficient of 0.887, indicating adequate reliability [41].

### 2.7. Data Collection Procedure

Ethical clearance was granted by the University of Cape Coast Institutional Review Board (Ref: UCC/IRB/CHAS/2023/03). Researchers obtained permission from the headmistress of Aburi Senior High School. After agreeing on the timing and duration for data collection, they introduced themselves to the respondents and explained the study’s objectives. The questionnaire was then distributed taking approximately 20–30 min to complete, with data collection lasting two weeks. Informed consent was obtained through consent forms, assuring respondents that participation was voluntary and that they could withdraw at any time. For participants under 18 years, a waiver of parental consent was approved due to minimal risk and impracticality in obtaining it. Respondent information was kept confidential and not shared with third parties. All procedures adhered to international research ethics guidelines, including the Declaration of Helsinki.

### 2.8. Data Analysis

The questionnaires were analyzed using IBM SPSS version 21. Descriptive analysis was used to analyze respondents’ background characteristics. The Pearson correlation coefficient test evaluated the relationship between respondents’ knowledge and practice of BSE. Logistic regression (likelihood ratio tests (LRT)) analysis was performed to determine the demographic predictors of BSE practice. All statistical tests were two‐sided, with a significance level of *α* = 0.05. Results with *p* < 0.05 were considered statistically significant.

## 3. Results

All the 385 distributed questionnaires were received representing 100% response rate. The results are presented in the following order: demographic characteristics of the respondents, knowledge on BSE amongst students, correlation between knowledge and practice of BSE, and demographic predictors of BSE.

### 3.1. Demographic Characteristics

The findings of the study on the demographic characteristics of the respondents are reported in Table 1.

**TABLE 1:** Demography of the respondents.

	Frequency	Percent (%)
Age		
10–17 years	105	27.3
18–22 years	280	72.7
Level		
Form 1	31	8.1
Form 2	129	33.5
Form 3	225	58.4
Religion		
Christian	285	74.0
Muslim	100	26.0
Knowing someone diagnosed of breast cancer		
No	157	40.8
Yes	228	59.2
Family history of breast cancer		
No	352	91.4
Yes	33	8.6
Attendance of conference, seminar, or workshop on breast cancer		
No	126	32.7
Yes	259	67.3

In Table 1, it was identified that the majority of the respondents (72.7%) were aged 18–22 years, while the remaining 105 (27.3%) were in the 23–29 years bracket. It was found that 58.4% were in Form 3, 33.5% in Form 2, and 8.1% in Form 1. Seventy‐four percent were (74.0%, 285) identified as Christian, while 26.0% (100) were Muslims. The responses of the respondents showed that more than half (59.2%, 228) knew someone who had been diagnosed with breast cancer, while 40.8% (157) did not know anybody with breast cancer. Additionally, 91.4% (352) confirmed that they did not know someone in their family diagnosed with breast cancer, whereas 8.6% (33) indicated they had a family history of breast cancer. The majority of the respondents (67.3%, 259) affirmed that they had attended a conference, seminar, or workshop on breast cancer, while 32.7% (126) denied attending such an event.

### 3.2. Knowledge of BSE Amongst Students

The data in Table 2 present mean scores and standard deviations for knowledge items related to BSE. Overall, participants demonstrated moderate to high knowledge levels, with mean scores ranging from 1.00 to 1.92 on a likely Likert scale. The highest knowledge was observed for using the middle fingertip pad to perform palpation with varying pressure (mean = 1.91, SD = 0.280) and maintaining skin contact during palpation (mean = 1.92, SD = 0.272), indicating strong awareness of tactile techniques. Knowledge was also high for assessing nipple abnormalities (mean = 1.91, SD = 0.280) and skin changes (mean = 1.83, SD = 0.377). However, understanding of specific positional techniques, such as orienting for the right breast examination (mean = 1.41, SD = 0.493) and visual inspection from multiple angles (mean = 1.57, SD = 0.495), was comparatively lower. The lowest score (mean = 1.00, SD = 0.000) was for palpation of the outer breast half, suggesting either universal knowledge or a potential limitation in the scale. The small standard deviations indicate consistent responses, but targeted education on positional techniques and comprehensive palpation methods could enhance BSE proficiency.

**TABLE 2:** Knowledge of BSE.

	Mean	Std. deviation
The breast self‐examination begins with tactile assessment as the patient searches for irregularities through palpation	1.75	0.436
To examine the right breast, the patient should orient herself by rolling on her left side and placing her right hand, palm up, on her forehead	1.41	0.493
A visual survey of the breast tissue requires an inspection from three angles, with arms at the side, arms raised above the head while bending forward, and hunched over with the hands placed on the hips	1.57	0.495
In breast self‐examination, the skin should be appraised for any rashes, erythema, puckering, dimpling, or textural anomalies resembling an orange peel	1.83	0.377
The middle fingertip pad should be used to perform small circles with light, medium, and deep pressure investigating varying depths of breast tissue	1.91	0.280
To complete the examination of the breast’s outer half, up and down motions of palpation are performed medially from the axilla to the nipple and vertically from the clavicle to just below the bra line	1.00	0.000
As the fingers traverse the breasts, they must remain in contact with the skin to avoid missing any tissue plane	1.92	0.272
Assessment of the inner half of the breasts requires changing to a supine position, removing the hand from the forehead, and placing the inactive arm at a right angle on the examination surface	1.75	0.435
The nipples must be monitored for scaling, erythema, pruritus, edema, discharge, or new inversion. Asymmetric venous distribution or dilation should also prompt further consideration	1.91	0.280

**FIGURE 1:**
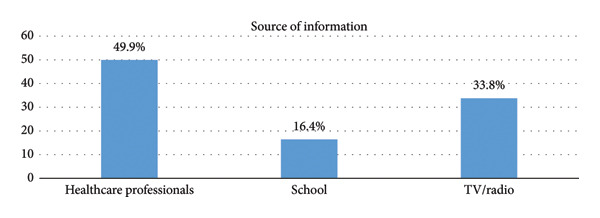
Sources of information of breast self‐examination.

All respondents reported being aware of breast cancer and obtaining information from different sources. Healthcare professionals were identified as the primary source of information (49.9%), followed by television and radio (33.8%) and schools (16.4%). This distribution is depicted in Figure 1.

### 3.3. Attitude Towards BSE Amongst Students

The analysis of attitudes towards BSE in Table 3 reveals significant insights into respondent’s attitude regarding this critical health practice. The mean score of 2.75 for the statement “doing BSE makes me feel so funny” indicates that many adolescent girls experience discomfort or awkwardness when considering BSE, which is further corroborated by the higher mean of 2.83 for “BSE will be embarrassing to me.” This suggests that social stigma and personal embarrassment play a substantial role in deterring adolescent girls from regularly performing BSE. However, the lower mean score of 1.41 for “doing BSE is wasting time” reflects that most participants do not perceive BSE as a futile endeavor; rather, they recognize its potential importance in breast health. This is an encouraging sign, especially when juxtaposed with the mean score of 2.33 for “feel uncomfortable, cannot do BSE once in a month,” indicating that while discomfort exists, it does not necessarily preclude adolescent girls from recognizing the value of BSE. Furthermore, the statement “if there is a lump, I prefer to get treatment from a traditional healer” scored a mean of 1.50, suggesting a preference for conventional medical treatment over traditional remedies, which may reflect an increasing awareness of the importance of seeking medical intervention when it comes to breast health concerns. The high mean score of 3.32 for “always search for information regarding BSE from the internet, magazine, and newspaper” underscores the proactive approach many adolescent girls take in educating themselves about BSE, indicating a desire for knowledge despite the associated discomfort. Additionally, a mean score of 2.66 for “discuss with my friends about BSE” suggests that while discussions about BSE do occur, they may not be as widespread or open as necessary to foster a supportive environment.

**TABLE 3:** Attitudes towards BSE.

	Mean	Std. deviation
Doing BSE makes me feel so funny	2.75	1.178
BSE will be embarrassing to me	2.83	1.470
Doing BSE is wasting time	1.41	0.493
Doing BSE makes me feel unpleasant	2.16	1.146
If there is lump, I prefer to get treatment from a traditional healer	1.50	0.501
Feel uncomfortable, cannot do BSE once in a month	2.33	0.948
All women should do BSE		
I really care about my breasts	2.08	0.494
I am not afraid to think about the breast cancer	2.93	1.328
Avoid BSE because I worry about having breast cancer	1.57	0.857
Interested in doing BSE	2.18	1.216
Always search for information regarding BSE from the internet, magazine, and newspaper	3.32	1.381
Discuss with my friends about BSE	2.66	1.031

### 3.4. Practice of BSE Amongst Students

The findings in Table 4 revealed that majority of the respondents (67.3%) knew how to perform BSE whiles the remaining 32.7% denied that they know how to perform the procedure. However, this awareness does not translate into a consistent understanding of best practices. For instance, only 25.20% of the respondents correctly identified that BSE should be performed on the same day each month, suggesting a lack of adherence to recommended guidelines for regularity. Conversely, a strong majority, 75.30%, accurately recognized that BSE is a monthly self‐check for changes or lumps, reflecting a general understanding of its purpose. Additionally, 74.80% of respondents acknowledged that BSE should be performed 5–7 days after menstruation, indicating awareness of the optimal timing for the examination.

**TABLE 4:** Practice of BSE.

	Yes (%)	No (%)
Do you know how to perform breast self‐examination?	259 (67.3)	126 (32.7)
The breast self‐examination should be carried out the same day each month?	97 (25.2)	288 (74.8)
Breast self‐examination is a monthly check‐up that women perform by themselves to check for any changes or breast lumps?	290 (75.3)	95 (24.7)
Breast self‐examination should be carried out 5–7 days after mensuration?	288 (74.8)	97 (25.2)

**FIGURE 2:**
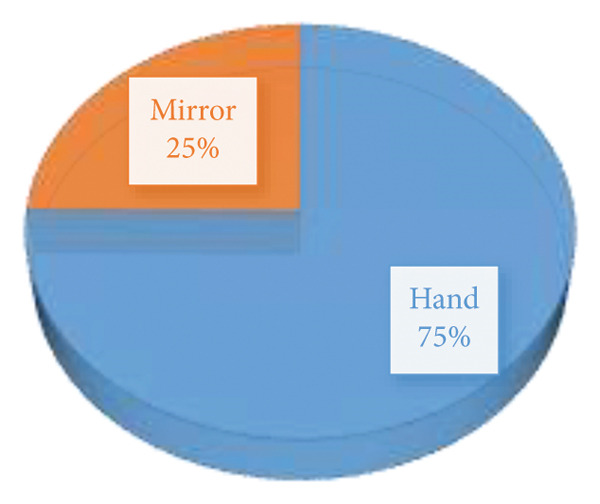
Materials used to perform breast self‐examination.

The results from Figure 2 indicate that 75% of the respondents identified the hand as one of the articles used to perform BSE and the remaining 25% added the mirror as another article used for BSE.

**FIGURE 3:**
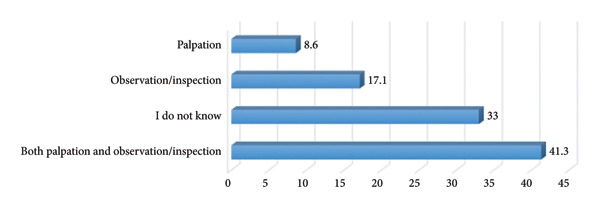
Appropriate procedure for breast self‐examination.

As shown in Figure 3, almost half of the respondents (41.3%) identified the use of both palpation and observation/inspection as the appropriate procedure for BSE. More than one‐tenth (17.1%) identified only observation/inspection and 8.6% stated only palpation as the appropriate procedure. However, more than a quarter (33%) stated that they do not know the appropriate procedure for performing BSE.

**FIGURE 4:**
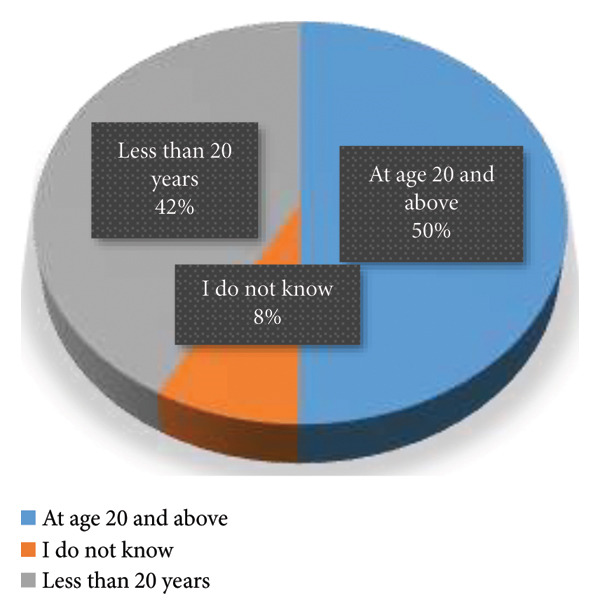
Appropriate age to begin breast self‐examination.

The responses to the appropriate age to begin BSE are illustrated in Figure 4. It was observed that half of the respondents correctly stated that the appropriate age was 20 years and above whiles 42% wrongly reported that the appropriate age was less than 20 years and 8% did not know the appropriate age.

### 3.5. Correlation Analysis Between Knowledge and Practice of BSE

From Table 5, the correlation analysis between knowledge and practice in the given data shows a statistically significant positive relationship (*r* = 0.149, Sig. = 0.003, *N* = 385). The weak but positive correlation (*r* = 0.149) suggests that as knowledge increases, practice tends to increase as well, although to a small extent. The significance level of 0.003, below the 0.01 threshold, indicates that this relationship is statistically significant, implying a low likelihood of chance occurrence. Therefore, we conclude that there is a meaningful, albeit weak, positive association between knowledge and practice among the study participants.

**TABLE 5:** Pearson correlations between knowledge and practice.

	Knowledge	Practice
Knowledge		
Pearson correlation	1	0.149^∗∗^
Sig. (2‐tailed)		0.003
N	385	385
Practice		
Pearson correlation	0.149^∗∗^	1
Sig. (2‐tailed)	0.003	
N	385	385

^∗∗^Correlation is significant at the 0.01 level (2‐tailed).

### 3.6. Sociodemographic Predictors of Practice of BSE

Logistic regression (LRT) was performed to test the influence of demographic factors on the practice of BSE. The LRT determines whether variables or parameters significantly improve or predict the model’s ability. Table 6 provides a detailed analysis of the significance and contribution of each predictor variable in the logistic regression model.

**TABLE 6:** Likelihood ratio tests.

Effect	Model fitting criteria	Likelihood ratio tests (practice of BSE)		
	− 2 log likelihood of reduced model	Chi‐square	Df	Sig.
Intercept	96.466^a^	0.060	5	1.000
Age	229.238^a^	132.833	5	0.000
Level	308.395^a^	211.989	5	0.000
Religion	96.744^a^	0.338	5	0.997
Know someone diagnosed of breast cancer	185.732^a^	89.327	5	0.000
Family history of breast cancer	98.328^a^	1.923	5	0.860
Conference/seminar/workshop attendance	361.053^a^	264.648	5	0.000

^a^Significance level *α* = 0.05.

The intercept‐only model shows that the −2 log likelihood value is 96.466, which serves as the baseline for evaluating the impact of adding the individual predictor variables to the model. Adding the “age variable” significantly improves the model fit, with a chi‐square value of 132.833 and a *p*‐value less than 0.001 (*χ* ^2^ = 132.833, *p* < 0.001). This indicates that age is a strong and statistically significant predictor in the model, enhancing the model’s ability to explain the variation in the outcome. Similarly, adding the “level variable” also leads to a significant improvement in model fit, with a chi‐square value of 211.989 and a *p*‐value less than 0.001 (*χ* ^2^ = 211.989, *p* < 0.001). This suggests that the level of the respondents is another important predictor in the model, contributing significantly to the overall explanatory power of the logistic regression.

In contrast, adding the “religion variable” does not significantly improve the model fit, as indicated by the nonsignificant *p*‐value of 0.997 (*χ* ^2^ = 0.338, *p* > 0.001). This implies that religion does not meaningfully contribute to the prediction of the outcome, after accounting for the effects of the other predictors in the model. On the other hand, the variable “know someone diagnosed with breast cancer” significantly improves the model fit, with a chi‐square value of 89.327 and a *p*‐value less than 0.001 (*χ* ^2^ = 89.327, *p* < 0.001). This indicates that this variable is an important predictor in the model and explains a substantial portion of the variation in the outcome. Adding the “family history of breast cancer” variable does not lead to a significant improvement in model fit, with a *p*‐value of 0.860 (*χ* ^2^ = 1.923, *p* > 0.001), suggesting that this variable is not a strong predictor in the model. Finally, adding the “conference/seminar/workshop attendance” variable results in a significant improvement in model fit, with a chi‐square value of 264.648 and a *p*‐value less than 0.001 (*χ* ^2^ = 264.648, *p* < 0.001). This suggests that this variable is a highly influential and significant predictor in the model, contributing considerably to the overall explanatory power of the logistic regression.

## 4. Discussion

### 4.1. Knowledge of BSE Amongst Students

BSE is crucial for early breast cancer detection, particularly in areas lacking advanced screening technologies like mammography. Understanding the KAP related to BSE among young women, especially senior high school students, is essential for developing effective education and prevention strategies. This study showed varying levels of knowledge of BSE among students. Research in Ghana and Nigeria indicates that while BSE awareness is relatively high, knowledge of correct techniques and frequency remains low [29, 42]. This contrasts with Segni et al. [43], who found only 8.7% of students in Ethiopia had good knowledge of BSE. Another Ethiopian study reported that just over 30.25% had good knowledge level of BSE [12]. Prakash et al. [44] found that 94.2% of female adolescents in Nepal had poor knowledge of BSE. Sarker et al. [45] noted a very low (34%) correct rate of BSE among female university students in Bangladesh. The current study’s higher knowledge levels may result from time and effective breast cancer awareness campaign in Ghana. Students primarily obtain BSE information from healthcare professionals, the media (TV/radio), and school, with healthcare professionals being the main source. Studies show that TV/radio and healthcare professionals are trusted sources of BSE information [46]. Fondjo et al. [9] found that students receiving BSE information from healthcare providers and the media are more likely to practice it regularly. Moreover, school‐based activities are significant sources of BSE information, with Nigerian studies indicating that students receiving formal education on BSE exhibit higher knowledge and practice levels [47]. These findings highlight the need to integrate various information sources, including formal education, media, healthcare professionals, and peer influence, to effectively promote BSE understanding and practice among senior high school students.

### 4.2. Attitude Towards BSE Amongst Students

The findings on students’ attitudes towards BSE indicate that some respondents feel discomfort and embarrassment when performing BSE and view it as unnecessary or inconvenient. These results align with Udoh et al. [48] and Afaya et al. [49], who identified cultural and personal sensitivities as significant barriers to BSE among young women. Emotional discomfort may deter regular practice. Afaya et al. [49] confirmed that although awareness of BSE is generally high, discomfort and fear often impede consistent practice. The sentiment that “BSE makes me feel unpleasant and uncomfortable; I cannot do BSE once a month” reflects that while some recognize the importance of BSE, psychological barriers hinder regular engagement. Research by Hussein et al. [26] similarly indicates that women acknowledge the benefits of BSE but often fail to incorporate it into their routine due to discomfort, forgetfulness, or lack of motivation. A significant cultural aspect arises from the statement, “if there is a lump, I prefer to get treatment from a traditional healer.” The low mean score for this suggests that most adolescent girls favor medical intervention over traditional treatments, contrasting with previous studies where reliance on alternative medicine was more prevalent, particularly in rural settings [50]. This finding underscores the need for continued health education to encourage appropriate medical care when abnormalities are detected. Fear of breast cancer also influences BSE practices, but the mean score for “avoid BSE because I worry about having breast cancer” suggests that fear may not be the primary reason for avoidance. Conversely, the high mean score for “I am not afraid to think about breast cancer” indicates that while respondents are aware of the disease, some still experience anxiety regarding their own risk. This finding corroborates with studies showing that fear of a positive diagnosis leads to avoidance behavior [51]. Asuming‐Bediako [52] identified fear of discovering a cancerous lump as a key reason women do not perform BSE regularly. Additionally, the mean scores suggest that young adolescent girls discussed BSE with peers, reflecting moderate engagement in breast health discussions. The role of peer education in increasing awareness and promoting preventive behaviors among adolescents is well‐documented [53]. A study in Nigeria by Ogunkayode and Ajuwon [46] revealed that while 56.0% of students had a positive attitude towards BSE, only 29.0% had ever practiced it. These findings emphasize the need for targeted educational interventions to demystify BSE and reduce stigma. By fostering open discussions and providing accessible information, healthcare providers can empower adolescent girls to incorporate BSE into their health routines, promoting early detection of breast cancer and enhancing overall awareness and proactive breast health management, ultimately improving health outcomes for women.

### 4.3. Practice of BSE Amongst Students

The majority of adolescent girls knew how to perform BSE, supporting Amegbedzi et al. [54], who found that 80% of respondents were aware of BSE. In contrast, Paruchuri et al. [55] reported that 72.5 of female university students did not perform BSE. Ibitoye and Thupayegale‐Tshwenegae [47] found that over 60% were aware of breast examination before education, rising to 91.7% after training. Similar studies confirm that BSE practice is generally low among various populations [9, 53, 56, 57]. Barriers may include lack of confidence, fear, perceived low breast cancer risk, and difficulties accessing screening and treatment [53]. Although many respondents knew how to perform BSE, the gap between this knowledge and the importance of regular practice highlights the need for improved educational efforts. These findings emphasize the necessity for targeted education to ensure adolescent girls understand both how to perform BSE and the significance of consistent practice for effective breast health monitoring.

The study found that 75% of respondents identified the hand as a key tool for performing BSE, while 25% recognized the mirror as important. This aligns with previous studies highlighting both palpation (using the hand) and visual inspection (using a mirror) in effective BSE execution [58, 59]. According to the American Cancer Society [60], BSE should involve both manual examination to detect lumps or texture changes and visual inspection in front of a mirror to identify skin dimpling, nipple retraction, or asymmetry. While a quarter of respondents recognized the mirror’s importance, more awareness is needed to reinforce comprehensive self‐examination practices. Regarding the procedure for performing BSE, 41.3% of respondents correctly identified that both palpation and observation/inspection are necessary. This reflects a positive level of awareness, though it falls short of optimal knowledge levels. Women who use both methods are more likely to detect abnormalities early compared to those who rely on only one [61]. However, 17.1% believed observation alone was sufficient, while 8.6% considered only palpation necessary. These misconceptions could lead to inadequate examinations, potentially missing early signs of breast cancer. Additionally, 33% reported not knowing the appropriate procedure for performing BSE. Similar knowledge gaps have been documented in studies from low‐ and middle‐income countries, where BSE education and breast cancer awareness programs are limited [55, 62]. These findings highlight the need for targeted educational interventions to improve knowledge and ensure women understand the correct techniques for BSE. Half of the participants correctly stated that BSE should commence at 20 years and above, as recommended by the American Cancer Society [60] and the World Health Organization [63]. However, 42% incorrectly believed BSE should start before age 20, while 8% admitted not knowing the appropriate age. The misconception that BSE should begin before 20 is commonly found in similar studies [12, 54]. While younger women are encouraged to develop breast awareness, routine BSE is not generally recommended before 20 years unless there is a family history of breast cancer [64].

### 4.4. Correlation Analysis Between Knowledge and Practice of BSE

The correlation between knowledge and practice of BSE is statistically significant and positive. Research consistently reports a positive correlation, though its strength varies. For example, a study in Nigeria and Saudi Arabia found a significant but low positive correlation (*r* = 0.242) between knowledge and BSE practice among female workers [65, 66]. Lafiaji‐Okuneye et al. [67] revealed a significant association (*r* = 0.234) between knowledge of BSE and practice, indicating that knowledge influences practice, but other factors may also play a role. A study in Bangladesh found a moderate correlation (*r* = 0.54) between knowledge and practice, suggesting that well‐informed students were more likely to engage in BSE [45]. The variability in these correlations highlights the influence of external factors such as cultural beliefs, access to health education, and supportive environments for BSE. For instance, Sarker et al. [45] reported that in schools with comprehensive health education, the correlation between knowledge and practice was higher, emphasizing the role of education in reinforcing health behaviors. Conversely, in settings where BSE is not promoted or where cultural stigma exists, the correlation tends to be weaker [68].

### 4.5. Sociodemographic Predictors of Practice of BSE

The study examined demographic factors influencing BSE practice among students. Results showed that age, educational level, knowledge of someone with breast cancer, and attendance at health‐related events were significant predictors of BSE practice. These findings support previous studies by Dinegde et al. [56] and Ben El‐Fakir et al. [57], which found that older students were more likely to engage in BSE due to increased awareness as they approach adulthood. However, Lafiaji‐Okuneye et al. [67] reported no association between age and BSE practice. Kebede et al. [69] found that secondary school students demonstrated better knowledge and practice, likely due to cumulative health education over time, suggesting that educational progression enhances health literacy and preventive behaviors. Personal connections to breast cancer, such as knowing someone diagnosed with the disease, further motivate students to practice BSE regularly. Kebede et al. [69] reported that students who knew someone diagnosed with breast cancer were more determined to practice BSE, partly due to a heightened sense of vulnerability. Participation in health‐related events also positively influences BSE practice. Women with adequate health literacy were more likely to engage in breast cancer screening [70], underscoring the importance of targeted educational interventions. On the contrary, religion and family history of breast cancer did not predict BSE practice. Solikhah et al. [71] found that Christian women had a significantly lower breast cancer screening rate than women from other religions, contradicting previous studies on the influence of religion. In this study, family history was not found to predict BSE practice, though Mihret et al. [40] found that students with a family history of breast cancer were significantly more likely to engage in regular BSE. Other systematic reviews supported the association between family history of breast cancer and BSE practice [72, 73]. The difference in findings may be due to the lower percentage of students reporting a family history of breast cancer (8.6%) in the current study. Teaching BSE should be intensified starting from high school, focusing on practice and its benefits for early detection of breast cancer. These findings emphasize the need to develop and promote educational intervention programs aimed at increasing breast cancer and BSE awareness in Ghana.

## 5. Conclusion

This study highlights gaps in BSE KAP among adolescent girls in the Eastern Region of Ghana. While awareness of BSE is relatively high, misconceptions about techniques, timing, and the use of both palpation and visual inspection persist. Emotional and cultural barriers such as discomfort and fear limit regular practice despite recognition of BSE’s importance. Key sources of information include peers, healthcare professionals, school‐based education, and media, indicating the need for multifaceted health education approaches. The positive correlation between knowledge and practice, along with factors like age, education level, prior exposure to breast cancer, and participation in health‐related events, demonstrates the importance of targeted interventions that strengthen health literacy and motivate consistent BSE behavior. Although religion and family history did not significantly predict practice in this study, variability in other contexts suggests that culturally nuanced strategies are essential. The findings reinforce the need for age‐appropriate, culturally sensitive, and school‐based breast health education programs to build confidence, address misconceptions, and promote regular BSE as a vital early detection strategy for breast cancer among Ghanaian adolescent girls.

## Author Contributions

Stella Sagoe and Sussana Sagoe contributed to the conceptualization, design, acquisition of data, and drafting the article. Patricia Tsotsoo Clottey and Thywill Amenuveve Degley supported the acquisition of data. Isaac Nyarko Kwakye and Daniel Adom‐Fyn conducted the analysis and interpretation, drafted the article, and revised it critically for important intellectual content. Emmanuel Lamptey assisted with the conceptualization and design. Ruth Nimota Nukpezah assisted with drafting the article and revising.

## Funding

The study did not receive any external funding.

## Disclosure

This work was carried out in collaboration among the authors. All authors have read and approved the final version of the manuscript. The corresponding author had full access to all of the data in this study and takes complete responsibility for the integrity of the data and the accuracy of the data analysis.

## Ethics Statement

Ethical clearance was obtained from the University of Cape Coast Institutional Review Board (Ref: UCC/IRB/CHAS/2023/03), and written consent form was signed before completing the questionnaire. The lead author, Stella Sagoe, affirms that this manuscript is an honest, accurate, and transparent account of the study being reported; that no important aspects of the study have been omitted; and that any discrepancies from the study as planned have been explained.

## Conflicts of Interest

The authors declare no conflicts of interest.

## Data Availability

The data that support the findings of this study are available from the corresponding author upon reasonable request.
